# The need to promote behaviour change at the cultural level: one factor explaining the limited impact of the *MEMA kwa Vijana* adolescent sexual health intervention in rural Tanzania. A process evaluation

**DOI:** 10.1186/1471-2458-12-788

**Published:** 2012-09-14

**Authors:** Daniel Wight, Mary Plummer, David Ross

**Affiliations:** 1 Medical Research Council Social and Public Health Sciences Unit, 4 Lilybank Gardens, Glasgow, G12 8RZ, UK; 2 London School of Hygiene and Tropical Medicine, 1 Keppel Street, London, WC1E 7HT, UK

**Keywords:** Behavioural interventions, Young people/adolescents, Sexual health, HIV/AIDS, Sub-Saharan Africa, Process evaluation, Culture, Tanzania, Structural barriers

## Abstract

**Background:**

Few of the many behavioral sexual health interventions in Africa have been rigorously evaluated. Where biological outcomes have been measured, improvements have rarely been found. One of the most rigorous trials was of the multi-component *MEMA kwa Vijana* adolescent sexual health programme, which showed improvements in knowledge and reported attitudes and behaviour, but none in biological outcomes. This paper attempts to explain these outcomes by reviewing the process evaluation findings, particularly in terms of contextual factors.

**Methods:**

A large-scale, primarily qualitative process evaluation based mainly on participant observation identified the principal contextual barriers and facilitators of behavioural change.

**Results:**

The contextual barriers involved four interrelated socio-structural factors: culture (i.e. shared practices and systems of belief), economic circumstances, social status, and gender. At an individual level they appeared to operate through the constructs of the theories underlying *MEMA kwa Vijana* - Social Cognitive Theory and the Theory of Reasoned Action – but the intervention was unable to substantially modify these individual-level constructs, apart from knowledge.

**Conclusion:**

The process evaluation suggests that one important reason for this failure is that the intervention did not operate sufficiently at a structural level, particularly in regard to culture. Recently most structural interventions have focused on gender or/and economics. Complementing these with a cultural approach could address the belief systems that justify and perpetuate gender and economic inequalities, as well as other barriers to behaviour change.

## Background

In 2010, roughly 900,000 more people were newly infected with HIV than died of it, and for every one person who started HIV anti-retroviral treatment two more became infected [1], driving a treadmill of increasing demand for future treatment. Although anti-retroviral treatment has dramatically improved and extended lives, unpredictable funding, weak health systems, and problems with side effects, adherence, and resistance mean that prevention remains crucial [2,3], as it is does with respect to other sexually transmitted infections (STIs) and unwanted pregnancies (e.g. [4,5]).

Since the identification of HIV/AIDS, a vast number of programmes have been implemented in sub-Saharan Africa to reduce sexual risk behaviours. Few have had rigorous outcome evaluations, and those have largely relied on reported behaviour measures. However, in recent years seven trials of behavioural sexual health programmes have used HIV as an outcome [6]. Unfortunately they all show that, despite improvements in knowledge and sometimes attitudes and reported behaviour, there were no improvements in biological outcomes, with one exception where herpes simplex virus 2, but not HIV, was lower in the intervention group [7].

One of the most rigorous evaluations was that of the multi-component *MEMA kwa Vijana* programme. This was implemented in rural Mwanza, northern Tanzania, which is ethnically 90% Sukuma, where predominant reliance on small-scale farming and fishing, plus some jobs in nearby towns or gold mines, make it fairly typical of rural sub-Saharan Africa. A large randomized controlled trial found no improvements in a range of biological outcomes [8,9]. The trial was complemented by a large-scale, primarily qualitative, process evaluation.

This paper attempts to explain the trial outcomes by reviewing the process evaluation findings. We first identify the main socio-cultural barriers to sexual behaviour change and then the main socio-cultural facilitators. We discuss the implications of these for the intervention’s underlying theories, argue for an ecological model which recognizes the role of structural factors, including culture, and recommend future behavioural interventions.

### The *MEMA kwa Vijana* programme and trial

The *MEMA kwa Vijana* programme emerged from British epidemiologists’ concern to rigorously evaluate methods of HIV prevention. It was developed and piloted over two years by Angela Obasi and Tanzanian colleagues, drawing on local studies (e.g. [10]) and similar interventions in the region, in consultation with education authorities [11,12]. The programme consisted of four components: limited community mobilization; a teacher-led, peer-assisted curriculum of 10–15 40-min sessions per year for the three upper years of primary school (http://www.memakwavijana.org); training and supervision of health facility clinicians to encourage youth friendliness; and the marketing of subsidised condoms by out-of-school youth [11,12]. It aimed to reduce the incidence of HIV, other STIs and unwanted pregnancy amongst young people, and to maximize sustainability by using existing government structures.

*MEMA kwa Vijana* was loosely based on Social Learning/Cognitive Theory [13] and the Theory of Reasoned Action [14]. Each identifies a set of component parts, or theoretical “constructs”, said to influence behaviour which are cognitive and largely overlap [15]. The main ones are knowledge, perceived risk/susceptibility, anticipated outcome/regret, self-efficacy/perceived control, intentions or goals, and subjective norms (individuals’ perceptions of the predominant views of appropriate behaviour). *MEMA kwa Vijana* addressed most of these constructs in multiple ways. For example, knowledge and perception of risks were promoted through teacher explanations and stories, class discussions, a drama serial performed by peer educators, and condom promotion. Pupils were encouraged to anticipate outcomes of their behaviour, and set goals for themselves, through reflective exercises. To promote self-efficacy, negotiation skills were developed through observing the drama serial and role playing, and condom use was demonstrated at health centres. Peer educators were expected to model behaviour and set new norms. Real and perceived impediments to condom access were addressed through class visits to health centers, where free condoms were available, and through promotion and distribution of condoms to out-of-school youth. These combined measures were meant to change goals or intentions, assumed in the Theory of Reasoned Action to be the key determinant of behaviour [16].

*MEMA kwa Vijana* was evaluated through a community randomized trial from 1998–2002 involving 9,645 young people [17]. Intervention participants had improved sexual health knowledge, more desirable reported attitudes, fewer reported sexual partners and greater reported condom use compared to controls [8]. Specifically, combined scores from three norms questions - whether girls could refuse sex with older men, with lovers, and after accepting a gift - improved from a very low base: at follow-up 22% of intervention males and 27% of females said girls could refuse. However, participants at the end of the trial showed no significant improvement in biological outcomes, in particular HIV, herpes simplex virus 2 and pregnancy, suggesting that reported behavioural data may have had poor validity [8]. In case more time was needed to detect an impact, a further survey was conducted in 2007/8 involving 13,814 15–30 year olds who had attended trial schools between 1999 and 2002. However, the findings were broadly similar to those earlier [9].

## Methods

The process evaluation methods - primarily participant observation, in-depth interviews and intensive group discussions - have been described in detail elsewhere [18], so only a brief summary follows. They were approved by the Tanzanian Medical Research Coordinating Committee and the London School of Hygiene and Tropical Medicine Ethics Committee.

Participant observation took place for a total of 158 person-weeks in nine villages, the four main villages being visited for approximately seven weeks per year from 2000–2002 by pairs of male and female researchers in their twenties. They lived with the family of a same-sex trial participant and accompanied him/her and peers in diverse daily activities.

Ninety-two trial participants were interviewed in-depth: 76 selected randomly, 13 because HIV-positive and three because pregnant while in school. Seventy-one were interviewed twice: in 1999/2000 and 2002. In addition, after two days of rapport building, six single sex groups of young villagers each participated in 3–4 discussions over several days, to address particularly sensitive topics, such as why girls have sex and the range of sexual activities practised.

Other data were collected through simulated patient visits to health clinics, group discussions with intervention pupils, observation of training sessions, internal monitoring and evaluation, and annual surveys of implementers [12,16].

The in-depth interviews, group discussions and simulated patient exercises were conducted in Swahili or Sukuma and audio-recorded. All the data, including participant observation notes, were translated into English and entered into NUD*IST. Five researchers coded them into 32 categories, partly pre-defined and partly emerging from the data, with checks for inter-rater reliability. Within categories information was summarized according to sub-themes. Provisional findings were tested through repeated readings of the summaries and transcripts and discussion with fellow researchers. Additional analysis often included reading everything relating to particular individuals. The massive dataset meant that, for a few topics, analysis was restricted to pre-selected portions.

## Results

### Implementation and engagement

Initial community mobilisation, mainly designed to reduce opposition, consisted primarily of a one-week visit to each ward for introductory meetings with parents and leaders, and the creation of a *MEMA kwa Vijana* advisory committee. Few refused to participate, but participant observation suggested that many adults knew very little about the purpose and content of the intervention, and some themes, in particular condom use, were controversial [12,16].

Process evaluation found that training courses for teachers, class peer educators, health workers and condom promoter-distributors were well implemented [11,12,16,19]. Almost all teachers delivered most sessions, some adopted interactive teaching styles and corporal punishment decreased. However, condoms could not be shown in primary school, leaving some pupils fundamentally confused, and some teachers continued non-participatory teaching, corporal punishment, or, occasionally, sexual abuse [12,16]. Class peer educators performed the drama serial well and generally understood the intervention content much better than their classmates. However, they could not answer complex questions and other pupils sometimes ignored or rejected their opinions [12,16].

Most classes visited a health facility as recommended, where condoms were demonstrated and presented as available free. However, participant observation suggested that adolescents remained very reluctant to use health services, fearing poor confidentiality and stigma. The simulated patient visits showed that, for those who did, provision was variable and limited, but of higher quality than in control communities [19].

Few pupils or other villagers had much awareness of *MEMA kwa Vijana* condom promoter-distributors, who were predominantly male volunteers elected by young villagers. Promoters reported difficulty selling condoms and were rumoured not to use condoms themselves, which was generally confirmed by their own accounts and those of their sexual partners. Due to the lack of demand for condoms, unsustainability, and cost, this intervention component was discontinued [20].

### Socio-cultural barriers to HIV prevention

Both the qualitative process evaluation and the biological trial outcomes showed that, despite the school component of *MEMA kwa Vijana* being delivered, and engaged in by pupils, largely as intended, sexual risk behaviours were not reduced. We will now try to explain this through the wider ethnography of young people’s lives. Although we can only outline the relevant findings from a vast quantity of data, fuller descriptions are provided elsewhere [12,18]. Here we identify the main contextual barriers to behaviour change, dividing them loosely between those relating to economics, social status and identity, and cultural beliefs. In practice they are highly interrelated, with gender cutting across them.

#### Economic-related barriers

##### Women’s lower social status and economic dependence on men

In the study villages men were generally more powerful than women, due to economic, kinship and normative factors. By and large, men had more access to paid employment and they owned the land and cattle. Women worked longer hours but largely in domestic work and subsistence farming, although some were able to earn money through cash crops or petty trading. The norms of wifely obedience, restricted gender roles, sexual double standards, and polygyny that disadvantage women across sub-Saharan Africa were also found in Mwanza [18].

Women’s subordinate status and very limited economic opportunities were fundamental barriers to sexual behaviour change. Girls generally received less schooling than boys and less intellectual stimulation to enable them to learn about sexual health, analyse their problems, and seek appropriate help. Girls’ socialisation to defer to their elders, in particular men, limited their self-efficacy to resist older men’s sexual advances, for instance teachers’. Their economic dependence on men meant some accepted unappealing marriages, married older men likely to have STIs, and/or were prevented from leaving husbands with extra-marital partners, or avoiding unsafe sex with them.

##### Sex as an important economic resource for women

Young women had far fewer economic opportunities than young men, so for most sex became a critical economic resource, encapsulated by an 18 year old woman: “What use is pleasure when there is no money?” [21]. Transactional sex underlay the great majority of non-marital relationships and was not, *per se*, perceived as immoral. In their teens girls were increasingly expected to provide for personal needs, such as soap, underwear, or food at school, but had very little access to cash. In stark contrast, they were repeatedly offered money or other gifts implicitly or explicitly in exchange for sex.

Yet transactional sex should not be seen only as “survival sex” among the poorest females; it was embedded in this culture at all income levels. While most young women engaged in it to meet basic material needs, many did so to seek beauty products, such as skin creams or “cool” clothes, or to accumulate business capital. This was reinforced by strong pressure to consume like their peers and to avoid ridicule: respectability and self-worth were dependent on receiving suitable recompense for sex. A 14 year old school girl described how a girl would be treated who received nothing for sex: **“**They will shout at her. They will laugh at her. … those girls will sit in groups just gossiping about her, and she will have no comfort, she will stay lonely”. Consequently enhancing young women’s income would relieve the pressure to engage in transactional sex, but would do little to erode the entrenched values around it. Transactional sex encouraged young women to start having sex and subsequently to change partners, since they could demand larger gifts for first sex within a relationship. It also discouraged condom use because many men would refuse payment. Although the *MEMA kwa Vijana* curriculum addressed transactional sex in several sessions, it did not recognise how fundamental the practice was both economically and culturally.

##### Poor quality of education and health services

At a broader level, Tanzania’s poverty meant that the education and health services, particularly in remote rural regions, were of very poor quality. Teachers themselves were often poorly educated and the best usually obtained urban jobs. Rural pupils had very low literacy, very limited knowledge of biology, and received little encouragement to think critically, hindering their engagement with *MEMA kwa Vijana*.

Many young women were misinformed and/or appeared ambivalent about pregnancy. Amongst those sexually active many believed conception was beyond their control, being determined by God or chance. Amongst those who actively tried to prevent pregnancy, ineffective traditional methods were widespread, while hormonal contraception was feared to risk permanent infertility: “some are said to have a rotting uterus after using contraceptive pills or injections for a long time.” (Female informant)

Basic understanding of HIV/AIDS was also still extremely poor, as found elsewhere in rural Africa [22,23]. Although *MEMA kwa Vijana* substantially improved pupils’ sexual health knowledge, many saw this as compatible with prior beliefs, for instance that traditional methods could prevent pregnancy or treat HIV/AIDS, some of which were harmful [24]. This illustrates the capacity for syncretic health beliefs widespread across sub-Saharan Africa [25].

Limited resources also meant health facilities often had inadequate staff training and chronic understaffing, with one-third of posts unfilled. Health centers were typically 3–10 kilometres away from homes and reached by foot. They were frequently poorly stocked and clinical advice could be inappropriate and/or not confidential. Those *MEMA kwa Vijana* participants who wanted to obtain modern contraception from health facilities feared criticism and stigma.

#### Social status/esteem-related barriers

##### Low status of youth

In rural Mwanza the generational hierarchy meant young people’s status was only just above that of pre-adolescent children, creating a particular barrier for a programme targeting youth. Their behaviour is unlikely to change if the beliefs of those around them not involved in the intervention, especially adults, remain unaffected. This was particularly relevant for schoolgirls with older sexual partners, and was apparent in peer educators’ difficulties promoting safer sex with other youth. Those not at school had marginal exposure to *MEMA kwa Vijana*, and many did not understand or support its goals. One head teacher reported that some parents: “… complain that the students are too young [and] that their children are being taught prostitution.” The long term evaluation of *MEMA kwa Vijana* suggests that eight years were not sufficient for the successive groups exposed to reach a critical mass and shift the predominant community norms [9].

##### Sexuality and masculinity

For all men, but particularly those not yet married or with children, sexual experience was a source of great pleasure, prestigious and central to their masculine identity. Young men often talked about sex, and sometimes fathers even bantered with their older sons about it, legitimating premarital sex and contravening cross-generational taboos against talking about sex. The ability to seduce desirable women was greatly admired by other men, resulting in persistent male pressure on young women, particularly those deemed “virtuous”. Young men were proud to be “strong” sexually, impotence was deeply humiliating, and impregnating partners was often welcomed, even out-of-wedlock, because it demonstrated fertility. Boys who had not had sex by age 15 were likely to be ridiculed, creating an important motive to start sex. A 19 year old man explained he first had sex at 11: “due to the pressure he was getting from youth who already had begun having sex. …his friends laughed at him, saying that he was afraid to seduce girls, or that he was impotent. He decided … to rid himself of the shame that confronted him every time he met his friends.”

Limited alternatives for young men to affirm masculine identity, such as through sports, disciplined employment, entrepreneurship, active fathering roles or becoming a patriarchal head of household, may have contributed to this emphasis on sexual prowess [26,27]. Young men’s sexual behaviour is unlikely to change without providing safer, credible ways to establish masculinity, to which females can relate.

#### Cultural barriers

##### Contradictory sexual norms, secrecy, rapid partner change, and concurrency

Young people grew up with contradictory sexual norms, discussed more fully elsewhere [18,28]. Some norms inhibited sexual activity, in particular that school pupils should be abstinent and that respectable young women should avoid pre-marital sex. Yet others condoned or encouraged sex, such as sex being biologically necessary for men or a resource for women to exchange. Young people managed these contradictory norms and expectations primarily by concealing their sexual relationships, especially from the older generation. As one fieldworker noted: “Young people have to hide [relationships] … because after parents discover …, they’ll expect [the boy] to marry her or to give her a lot of money.” For young women, concealment was important to avoid parental punishment and protect their sexual reputations, while both sexes “prefer to conceal their sexual relationship for fear of being discovered by their other lovers … to avoid quarrels which may ensue.” Although boys liked to indicate their sexual activity to peers, they often did so without naming their partners.

Such concealment of non-marital relationships is widespread elsewhere in Tanzania [29] and sub-Saharan Africa [30,31]. It meant they were not acknowledged by parents nor reinforced through the endorsement of families and peers, which might have promoted longer, more exclusive relationships. Instead clandestine relationships facilitated rapid partner change and concurrent relationships. Young people learned from an early age to present themselves differently as different social contexts required, and, importantly, to maintain discretion rather than strict adherence to moral norms [31].

Hiding sexual relationships was interlinked with a taboo against parent–child discussion of sexual issues, deemed improper and shameful, as has been widely documented elsewhere in sub-Saharan Africa (e.g. [32,33]). This meant parents rarely discussed sexual risks or endorsed sexual health programmes with their children.

##### Negative condom beliefs and attitudes

Very few villagers had experience with condoms, and among those who had, most had only used them once or twice. This was because of negative condom beliefs and concepts of what constitutes meaningful, natural, or pleasurable sex [18,34]. While the most widely expressed belief was that condoms reduce men’s sexual pleasure, expressed in metaphors such as “eating a sweet in its wrapper”, they were also associated with infection and infidelity. Many other reported worries may have been secondary rationalizations to disguise concern about physical pleasure. Furthermore, adults opposed discussing condoms with young people in case of promoting sexual activity. For the rare young people who may have considered using condoms, access was limited, especially for girls, with intermittent supplies, distant health facilities, and limited confidentiality.

It is a considerable achievement that *MEMA kwa Vijana* negotiated the discussion of condoms in schools and their demonstration in health facilities, and improved participants’ condom knowledge, attitudes and reported use. However, educational restrictions on depicting condoms meant many pupils and even peer educators only vaguely understood how they were used. Promoting condoms to prevent disease might have been more effective if it had emphasized the impact of STIs on fertility, given that almost all young people aspired to have children in their lifetimes.

##### Low salience of, and perceived susceptibility to, HIV/AIDS

Several factors contributed to the low prominence of HIV/AIDS in day-to-day life. For most rural young people material insecurity meant focusing on immediate daily concerns rather than a long-term threat: completing routine tasks, getting food, water and soap, and maintaining parents’ approval and peers’ respect. This was probably particularly the case for young people who lacked long-term educational, business, farming, or family goals. For those who understood it, the long duration between HIV infection and symptoms also dissipated immediate concern.

Most people could reduce their sense of personal risk by distancing themselves from those they perceived vulnerable to HIV/AIDS. Villagers generally thought it affected those living in towns and cities, particularly those with “immoral” sexual practices, and adults rather than young people. Furthermore, HIV/AIDS was largely invisible in rural areas, due to relatively low prevalence, very limited testing, poor health services and stigma. The increased mortality due to AIDS was often attributed to the specific opportunistic infection and/or witchcraft [35]. When close relatives or friends did know the root cause, AIDS was very rarely openly acknowledged.

##### Limited agency and short-term decision making

Economic and cultural factors reinforced each other to create a limited sense of personal agency, another important obstacle to behaviour change. Poverty and poor education led villagers to talk about their lives fatalistically, particularly the poorest, least-educated, and women, frequently attributing things to God’s will or “*bahati mbaya”* (Swahili for “bad luck”) [36]. This led to short-term decision-making, such as agreeing to get engaged or elope after a very brief meeting. In sexual encounters young people often focused on the immediate advantages of sex, rather than possible unwanted pregnancy or infection, even when these consequences were well known.

### Socio-cultural facilitators of successful prevention in Mwanza

#### Restrictive norms

As noted above, young people’s sexual culture was characterized by contradictory norms, some inhibiting sexual activity and others encouraging it [28]. The most important restrictive norms were that school pupils should be abstinent, young women should avoid overt pre-marital sex, married couples (particularly wives) should be faithful, and sex should only be discussed in particular social contexts. These norms constrained young women much more than young men and, as elsewhere in Tanzania [37], they were of far greater importance in restricting sexual behaviour than the threat of HIV infection. They made *MEMA kwa Vijana*’s messages to delay sex and be faithful culturally appropriate, and their protective potential should be explored elsewhere while being alert to unintended consequences, as found in Uganda [38].

#### Education and religion

Schooling protected against sexual risks in several ways, highlighting the potential of interventions to prolong formal education. Many parents were keen that pregnancy or early marriage should not waste the costs of their daughters’ schooling, and so they tried to prevent them until primary school was completed. The few young women at secondary school tended to have extraordinary parental support, creating a strong obligation to avoid pregnancy, while school boys generally had less money than their out-of-school peers to pay for sex. More generally, the small minority of youth with strong educational ambitions were more averse to sexual risk, perhaps because they were more future-oriented.

Growing up in a devout Christian or Muslim family promoted abstinence and fidelity, especially if the young shared their parents’ strong religious beliefs. When they did not, religious disapproval and the threat of punishment sometimes reduced their sexual activity, but sometimes it prompted greater secrecy. Religious beliefs could also inhibit the use of condoms, as found in Zambia [39]. These potential contradictory outcomes, resulting from intersections of religious doctrine, generational authority and sexual norms, should be taken into account in developing interventions with formal religions.

#### Parents’ concern for their children’s health and welfare

Parents in rural Mwanza were deeply concerned about the well-being and future prospects of their children [40], even though overt expressions of affection were unusual. Elsewhere in sub-Saharan Africa, parents’ close supervision of, and connection with, their children has been associated with children’s healthier behaviours [33,41-43]. Beyond love, parents had multiple incentives to protect their children’s health: to maximise bridewealth payments by restricting daughters’ sexual relationships; to continue their lineage, a core part of villagers’ identity; and to be cared for in old age. In terms of sexual health, parental concern was primarily expressed through trying to prevent sexual activity. Harnessing parental concern in other ways might make interventions more meaningful and effective.

## Discussion

### Theoretical implications

#### Social cognitive theory and theory of reasoned action

To what extent do these findings provide empirical support for Social Cognitive Theory and the Theory of Reasoned Action, which originally underlay *MEMA kwa Vijana*? As outlined in the Background, in these theories the main constructs hypothesised to shape behaviours are knowledge, perceived risk/susceptibility, anticipated outcome/regret, self-efficacy/perceived control, intentions/ goals and subjective norms [13,44]. These constructs, or similar ones [15], are also central to several other social psychological theories of behaviour change, such as the Information, Motivation, Behavioural skills Model [45], the AIDS Risk Reduction Model [46] and the Theory of Planned Behaviour [47], although the theories differ in how they represent the causal relationships between these constructs. Nearly all the contextual barriers to behaviour change identified above are likely to: obscure the potential negative outcomes of risky sexual behaviour; undermine intentions or goals to avoid such behaviour; undermine self-efficacy or perceived control to do so; and create or reinforce norms that encourage risk behaviours. Conversely, all the contextual facilitators identified above could, potentially, enhance awareness of risks and create or strengthen intentions to avoid them. Notably, they are either about protective norms or are likely to create or reinforce them.

The ethnographic findings therefore go some way to endorse the theories underlying the intervention, in that they point to the importance of several of their central constructs and show that when these do not change, behaviour does not change. However, in social psychological terms this is an extremely crude validation: the qualitative data clearly cannot quantify the relative importance of the constructs and, furthermore, they point to greater complexity about which constructs determine behaviour. More fundamentally, the original theories only partially explain why the intervention did not modify these constructs, or how this might be done [12]. It may be that we need a different theoretical perspective to do this, one suggested by recognizing that most of the barriers to, and facilitators of, behavioural change seem to operate through social norms. It is striking that - although responses to the three survey questions on norms improved by the end of the trial - only one quarter of the intervention group answered them in the desired way.

#### The social context of individual cognitions

Human life operates simultaneously at many different levels, and social phenomena are experienced by individuals in terms of individual cognitions or understandings. These are the focus of social psychological theories, which hope to adequately incorporate social factors through their effect on cognitions. Yet the focus on individual cognitions, and the assumption that they are the main determinant of behaviour, ignores that they are generated, maintained, and modified through social interaction, for instance between relatives, peers, and fellow villagers [48]. From a sociological perspective, the existence of individual cognitions completely independent of their social context has long been questioned [49].

Therefore, while behavioural interventions often try to modify participants’ attitudes and norms, any programme influence may be submerged by conflicting dominant social norms and practices [50]. This is a particular danger if the target group is of low social status, such as young people, and especially young women, in sub-Saharan Africa. As the authors of a recent systematic review of HIV behavioural prevention for young people concluded:

“Interventions, thus far, perhaps were not sufficiently cognisant of local community norms, be it concerning concurrency, condom use or other behaviours. Changing these community norms will require more than a behavioural intervention targeting individual youth; a shift in paradigm may be necessary, perhaps from a focus on the individual to a scenario of greater community responsibility.” [51]

#### Culture

Social norms do not, however, seem to adequately explain how several of the barriers to behavioural change operate. From a social anthropological perspective, social norms are expressions of “a larger map of local ideas and beliefs” [52], in short, culture. We use the term “culture” in Douglas’s sense to describe “a way of thinking that justifies a way of living.” [53]. For instance, most societies with systematic gender inequalities have elaborate cultural beliefs that perpetuate and justify them. In this way we consider culture to be structural, in that it constitutes an underlying pattern of the social system largely beyond individuals’control. In recent decades cultures have been recognized to be dynamic and subject to modification by interested parties, rather than static and homogeneous [53-55], but two essential features are that they are *shared* ways of thinking and are *systems* of beliefs.

Culture depends on interactions between people and is therefore best analysed at a collective level. Furthermore, the full significance of specific cultural beliefs can only be understood in relation to a wider system that gives them meaning and resonance: “the elements of a cultural system make sense only in relation to one another” [52]. Often those sharing the beliefs are not conscious of the wider belief system of which they are a part, just as most people are unaware of their language’s grammatical rules, hence the value of an outsider (“etic”) perspective to interpret culture. To take one example, young women’s belief that it is appropriate to request material exchange for sex will have emerged from, and be reinforced by, their own and others’ conversations and observations within their families, schools and villages. Further, the belief is related to a set of deeper cultural ideas that reinforce each other, in particular that access to sex is a valuable female resource, that female resources are exchanged for material through bridewealth, and, perhaps most fundamental, that gifts should be reciprocated.

In highlighting the significance of culture we should acknowledge that to understand more fully the strengths and limitations of an intervention of this kind, in the broadest sense, we should also examine the cultures of the researchers who initiated it, of the research and funding institutions involved, and of those who designed the programme [56]. There is not scope here to provide an ethnography of the research project itself, which might examine, amongst other things, how these cultures shaped the prioritisation of the trial versus intervention development, the initial evaluation design and what was funded, how the trial design evolved and the intervention’s implicit values, content and implementation. More details on the project are provided elsewhere [12,18] but an ethnography of the project itself would be a distinct study probably best conducted by someone outside the research team.

#### Other structural factors: economics, social status and gender

Douglas’s definition of culture [53] cleverly side-steps causation, but acknowledges that culture is intertwined with other social structures. With respect to the barriers and facilitators of sexual behaviour change in rural Tanzania, these structures are primarily economic and/or about social status and gender. Economic and gender factors have been widely analysed as structural drivers of the HIV epidemic (e.g. [57]), but few have recognized the importance of social status or culture. The role of economic factors should be apparent from the main findings. Social status, in the Weberian sense of the social honour attached to a social position, is especially important in small-scale communities, and underlies the relative powerlessness of young people and the importance of masculine identity. Concern with social status, and particularly respectability, is also probably central to how several of the facilitators of behaviour change operate. Educational success and religious devoutness both bring social honour, while restrictive norms, in particular for young women not to show an interest in sex, and conforming to village expectations, are both about maintaining respectability. Gender inequalities are interwoven with economic and social status factors, and are at the core of two of the barriers to behaviour change: women’s lower social status and sex being an economic resource for them.

These findings are therefore in keeping with an ecological model of health behaviour which emphasizes multiple levels of influence [58,59], commonly represented as a dissected onion with macro influences on the periphery and intrapersonal ones at the centre. A weakness of many ecological models is lack of specificity about which of the many acknowledged influences are most important [58]. Although the different influences are interdependent [60], our findings from rural Tanzania suggest that structural factors should receive more attention, in keeping with an analysis of the Sonagachi Project which concluded that health behaviours are most strongly influenced by structural forces [61]. In particular, our findings highlight the importance of cultural beliefs, which have received little recognition in the public health literature that draws on the ecological approach [59].

### Recommendations

#### Intervention at a cultural level

The broad implications of this interpretation for future behavioural sexual health interventions are twofold. First, interventions are most likely to be effective if multi-level: addressing individual factors but also collective and structural barriers to behaviour change [62,63]. Second, within these multi-level interventions we should specifically target the cultural level.

Working at a cultural level requires collective interventions, and acknowledging that cultural beliefs cannot be readily modified without addressing the wider systems of meaning of which they are part. For new norms to endure and generate different patterns of behaviour, they need to be confirmed and reinforced through social interaction: “… shared meanings are created and sustained – a process which happens, as it were, *between* people, not simply in their private thought worlds” ([25]: 11). Likewise Appadurai [52] highlights the importance of “rituals” of practice to establish internal consensus in cultural change. Therefore the broader the group targeted within a community, the more opportunity there is for such social interactions to occur, and their repetition sustains the impact of the original intervention.

Although there have been many critiques of how participatory approaches work in practice (e.g.[64,65]), genuine community participation can lead to interventions which involve a wide section of the population. Importantly, participatory interventions are likely to include, or at least to have consulted, local people with power. Many of the best examples of substantial and sustained sexual behavioural change are of groups that acted on their own initiative, such as Ugandan communities and gay communities in the USA early in the HIV/AIDS epidemic [66]. As Parikh concluded in her study of HIV and marriage in Uganda, “Risk-reduction strategies that build on a community’s resources, understandings, and needs have a greater chance of being sustainable and effective than ones that impose external concepts, assumptions and priorities” ([38]: 1206).

Ideally, programmers would use cultural elements that provide pre-existing motivations for behaviour change, as in Panter-Brick et al.’s call for “culturally compelling” interventions that address local priorities, and which communities feel they “own”. They use an example of locally-composed songs to encourage the mending of malaria bed nets in the Gambia, which achieved behavioural change because they “articulated pre-existing social priorities” and “community ownership of the songs was [their] salient factor” ([67]: 2823–4). With respect to HIV/AIDS, it has been suggested that the rich information on HIV in some popular Tanzanian songs gives them great potential for “culturally relevant and engaging” prevention messages ([68]: 1360).

In Mwanza the limited ethnographic data - particularly on sexuality - available when *MEMA kwa Vijana* was developed meant that the curriculum was not as culturally compelling as it might have been. Certain crucial aspects of sexual culture were not sufficiently addressed, such as rapid partner change and the central importance of transactional sex for girls. Furthermore, the programme did not make enough use of some things that *did* motivate young people. For example, being “*kisasa”* (modern or cool) could have been equated with low risk sexual behaviours, or the desire for money might have been exploited by cash transfers conditional on a girl staying in school, as successfully demonstrated elsewhere [69]. In the longer term, motivations such as safeguarding fertility could have been exploited to promote condom use for STI prevention. Extensive research to inform intervention development may seem inappropriate given the urgency of the epidemic, but without it interventions are less likely to utilize endogenous motivations in culturally appropriate ways.

However, there is an obvious tension between maintaining cultural compatibility and modifying those cultural beliefs that perpetuate unhealthy behaviours. Nevertheless, the dynamic, non-monolithic, sometimes contradictory nature of cultures offers the possibility of identifying existing cultural beliefs that can be exploited to modify others which are barriers to risk reduction. For instance, our findings suggest three important cultural goals for interventions, each of which go beyond specific norms to involve wider systems of belief.

1. Seek greater openness about sexual relationships so that risks can be discussed with parents, relationships can be strengthened through social endorsement, and partners become more accountable, which means tackling the culture of discretion. This might be pursued by exploiting parents’ widely expressed concerns about their children’s sexual health [40] and some young people’s ideal of monogamous relationships. Pursuing this recommendation might jeopardize the protective nature of restrictive sexual norms, described above, and so it would have to be implemented sensitively and with attention to unintended consequences.

2. Develop stronger relationships that precede unprotected sex, so that partners are more compatible and more able to resolve problems rather than change partners. Again, this might be pursued by exploiting ideals of longevity and fidelity in relationships, as demonstrated in Ugandan interventions [38,70].

3. Develop acceptable forms of masculinity for young men in which sexual experience is not a core component, for instance drawing on their love of sport, or roles as fathers [38,71].

#### Modifying other structural factors

The other broad implication of our interpretation is that interventions should operate at multiple levels. *MEMA kwa Vijana* combined four different components at the micro- and meso-levels, but not the macro-level. Today far more wide-ranging combinations of interventions are recommended, with multiple behavioural goals and which operate in multiple sectors [62,63]. However, interventions should not be adopted indiscriminately but should be tailored to the local epidemic, which requires understanding its underlying drivers and which are modifiable [72]. We hope that the research reported here, and in much greater detail elsewhere [18,12], contributes to such understanding for the epidemic in rural Tanzania.

Intervention combinations should include those with the strongest evidence of effectiveness, which, to date, are all bio-medical, including condoms, male circumcision, Human Papilloma Virus vaccination [73] and tenofovir vaginal microbicide gel [74]. They mainly operate at the individual level, though the use of condoms, and to some extent vaginal microbicides, depends on agreement or acquiescence by both sexual partners.

However, the non-cultural structural factors that underlie barriers to behaviour change, and which reinforce problematic features of the culture, should also be addressed within intervention packages. If not, changes of knowledge, norms, intentions, or skills at an individual level will have limited effect. Interventions that address both economic and cultural factors are likely to be most effective: cultural values and beliefs which seem entrenched tend to change if the economic factors with which they are entwined are modified [55]. Furthermore, addressing structural factors often means broadening the focus of interventions and overlapping other high-priority policies which should improve political and financial support.

Evidence of the effectiveness of structural level interventions is not as good as that for individual level interventions, in part because they are more difficult to rigorously evaluate. However, there is strong evidence for mass media broadcasts and education-entertainment [73,75] which operate at a cultural level. In relation to transforming gender roles, there is promising evidence that *Stepping Stones* can reduce key risk factors for HIV [7], although as delivered in that trial it seemed to operate through individual empowerment, rather than at a community level. There is less strong evidence for parenting programs [76].

Our findings suggest three important structural level goals for adolescent HIV prevention interventions in Africa beyond those directly addressing sexual culture.

1. Train and monitor teachers to: have supportive relationships with pupils, boost pupil confidence, encourage critical thinking, challenge dominant gender norms, and not engage in physical or sexual abuse.

2. Ensure that all young women complete primary school, even if they become pregnant, and the majority go on to secondary schooling, for example through conditional cash transfers [69].

3. Deliver income-generating or cash transfer schemes for young women to reduce their need for transactional sex and facilitate the selection of safer partners [69].

These goals, and the cultural ones outlined above, could be pursued through diverse intervention activities. Ideally the optimum modes of delivery would be identified through collaborations of practitioners, community members and researchers and then subjected to rigorous outcome evaluations, which should acknowledge the long time scales probably necessary to see effects.

## Conclusion

Ethnographic findings from a process evaluation of the *MEMA kwa Vijana* programme suggest that the barriers and facilitators of sexual behaviour change amongst young people in rural Tanzania operate, to an important extent, structurally. This supports an ecological model that acknowledges influence at several different levels. Our findings show that, at an individual level, social norms are extremely important, but these are elements of a broader culture which is shared, made up of systems of belief, and should be regarded as structural. Preventative interventions need to address behavior change at multiple levels simultaneously, including the oft-neglected but very important level of culture.

## Competing interests

The authors declare that they have no competing financial interests. However, having been involved in the development of the *MEMA kwa Vijana* intervention, they all, and in particular DR, had an intellectual, academic and personal interest in demonstrating the effectiveness of the intervention.

## Authors’ contributions

DW led the conception and design of the study, acquired funding (with DR and Richard Hayes), helped train the fieldwork team, analysed the data, drafted the manuscript and successively revised it in the light of co-authors’ and reviewers’ comments. MP made substantial contributions to conception and design, trained and supervised the fieldwork team, led the analysis and interpretation of data, drafted some sections of the manuscript and gave detailed intellectual criticism of successive versions. DR made substantial contributions to the conception and design, acquired funding (with DW and Richard Hayes), contributed to data interpretation, gave detailed intellectual criticism of successive manuscripts and was principal investigator of the *MEMA kwa Vijana* trial. All authors read and approved the final manuscript.

## Pre-publication history

The pre-publication history for this paper can be accessed here:

http://www.biomedcentral.com/1471-2458/12/788/prepub

## References

[B1] UNAIDS World AIDS Day Report 2011 Geneva: UNAIDS

[B2] Mills EJ Adherence to HAART: a systematic review of developed and developing nation patient-reported barriers and facilitators PLoS Med 2006 3 11 e438 10.1371/journal.pmed.0030438 17121449 PMC1637123

[B3] UNAIDS AIDS Epidemic Update 2009 Geneva: Joint UN Programme on HIV/AIDS

[B4] Juárez F Introduction to the special issue on adolescent sexual and reproductive health in sub-saharan Africa Stud Fam Plan 2008 39 4 239 244 10.1111/j.1728-4465.2008.00172.x 19248712

[B5] WHO Unsafe Abortion: Global and Regional Estimates of Incidence of Unsafe Abortion and Associated Mortality in 2000 2004 Geneva: World Health Organization

[B6] Ross DA Behavioural interventions to reduce HIV risk: what works? AIDS 2010 24 S4 S14 10.1097/01.aids.0000390703.35642.89 21042051

[B7] Jewkes R Impact of stepping stones on incidence of HIV and HSV-2 and sexual behaviour in rural South Africa BMJ 2008 337 a506 10.1136/bmj.a506 18687720 PMC2505093

[B8] Ross DA Biological and behavioral impact of an adolescent sexual health intervention in Tanzania: a community-randomised trial AIDS 2007 21 14 1943 1955 10.1097/QAD.0b013e3282ed3cf5 17721102

[B9] Doyle AM Long-term biological and behavioural impact of an adolescent sexual health intervention in Tanzania PLoS Med 2010 7 6 e1000287 10.1371/journal.pmed.1000287 20543994 PMC2882431

[B10] Matasha E Sexual and reproductive health among primary and secondary school pupils in Mwanza, Tanzania: need for intervention AIDS Care 1998 10 5 571 582 10.1080/09540129848433 9828954

[B11] Obasi AI Rationale and design of the MEMA kwa Vijana adolescent sexual and reproductive health intervention in Mwanza Region, Tanzania AIDS Care 2006 18 4 311 322 10.1080/09540120500161983 16809108

[B12] Plummer ML Promoting Abstinence, being Faithful, and Condom use with Young Africans: Qualitative Findings from an Intervention Trial in Rural Tanzania Lexington: In press

[B13] Bandura A Health promotion by social cognitive means Health Educ Behav 2004 31 2 143 164 10.1177/1090198104263660 15090118

[B14] Ajzen I Fishbein M Cliffs E Understanding Attitudes and Predicting Social Behaviour 1980 New Jersey: Prentice-Hall

[B15] Michie S Making psychological theory useful for implementing evidence based practice: a consensus approach Qual Saf Health Care 2005 14 1 26 33 10.1136/qshc.2004.011155 15692000 PMC1743963

[B16] Plummer M A process evaluation of a school-based adolescent sexual health intervention in rural Tanzania: the MEMA kwa Vijana programme Heal Educ Res 2007 22 500 512 10.1093/her/cyl103 17018767

[B17] Hayes RJ The MEMA Kwa Vijana project: design of a community randomised trial Contemporary Clin Trials 2005 26 430 442 10.1016/j.cct.2005.04.006 15951245

[B18] Plummer ML Wight D Young People's Lives and Sexual Relationships in Rural Africa: Findings from a Large Qualitative Study in Tanzania 2011 Lanham: Lexington

[B19] Larke N Impact of MEMA kwa vijana …on use of health services by young people J Adolesc Heal 2010 47 512 522 10.1016/j.jadohealth.2010.03.020 20970087

[B20] Terris-Prestholt F Costs of an adolescent sexual health program in Mwanza, Tanzania Sex Transm Dis 2006 33 10 S133 S139 16652070 10.1097/01.olq.0000200606.98181.42

[B21] Wamoyi J Transactional sex amongst young people in rural northern Tanzania: an ethnography of young women's motivations and negotiation BMC Reproductive Health 2010 7 2 10.1186/1742-4755-7-2 PMC2867784 20429913

[B22] Ezekiel MJ The constitution of antiretroviral therapy in local discourse among youth in Kahe Tanzania Soc Sci Med 2009 68 957 964 10.1016/j.socscimed.2008.12.016 19136190

[B23] Robins S Foot soldiers of global health: teaching and preaching AIDS science and modern medicine on the frontline Med Anthropol 2009 28 1 81 107 10.1080/01459740802640990 19184757

[B24] Plummer ML Young women's attempts to control their reproduction through contraceptive and fertility practices in rural Tanzania Tanzanian J Health Res 2010 12 3 178 193

[B25] Pool R Geissler W Medical Anthropology 2005 Berkshire: Open University Press

[B26] De Visser RO Smith JA Alcohol consumption and masculine identity amongst young men Psychol Heal 2007 22 595 614 10.1080/14768320600941772

[B27] Steinberg J Three Letter Plague 2009 London: Vintage

[B28] Wight D Contradictory sexual norms and expectations for young people in rural northern Tanzania Soc Sci Med 2006 62 4 987 997 10.1016/j.socscimed.2005.06.052 16139937

[B29] Setel PW A Plague of Paradoxes 1999 Chicago: University of Chicago Press

[B30] Nzioka C Perspectives of adolescent boys on the risks of unwanted pregnancy and sexually transmitted infections: Kenya Reprod Health Matter 2001 9 17 108 117 10.1016/S0968-8080(01)90014-X 11468825

[B31] Helle-Valle J Arnfred S Understanding sexuality in Africa: diversity and contextualized dividuality Re-thinking Sexualities in Africa 2004 Uppsala: Nordiska Afrikainstitutet 195 210

[B32] Phetla G Increasing women’s skills and motivation for sexual communication with young people in rural South Africa AIDS Educ Prev 2008 20 6 504 518 10.1521/aeap.2008.20.6.504 19072526 PMC3494081

[B33] Biddlecom A Awusabo-Asare K Bankole A Role of parents in adolescent sexual activity and contraceptive use in four African countries Int Perspect Sex Reprod Heal 2009 35 2 72 81 10.1363/3507209 19620091

[B34] Plummer M "Farming with your hoe in a sack": condom attitudes, access and use in rural Tanzania Stud Fam Plan 2006 37 1 29 40 10.1111/j.1728-4465.2006.00081.x 16570728

[B35] Mshana G AIDS and sexually transmitted infection causation beliefs in rural Mwanza, Tanzania Cult Heal Sex 2006 8 1 45 58 10.1080/13691050500469731 16500824

[B36] Desmond N Adaptation and resignation to socially constructed perceptions of risk in rural Tanzania. MRC SPHSU 2009 University of Glasgow

[B37] Dilger H Sexuality, AIDS, and the lures of modernity. 22:23–52 Med Anthropol 2003 22 23 52 10.1080/01459740306768 12641295

[B38] Parikh SA The political economy of marriage and HIV: the ABC approach, â€œSafeâ€□ infidelity, and managing moral risk in Uganda Am J Public Health 2007 97 7 1198 1208 10.2105/AJPH.2006.088682 17538057 PMC1913085

[B39] Agha S Hutchinson P Kusanthan T The effects of religious affiliation on sexual initiation and condom use in Zambia J Adolesc Health Off Publicat Soc Adolesc Med 2006 38 5 550 555 10.1016/j.jadohealth.2005.04.012 16635766

[B40] Remes P Community perceptions of adolescent sexual and reproductive health risks and appropriate interventions in rural Mwanza, Tanzania Cult Heal Sex 2010 12 3 279 292 10.1080/13691050903395145 19941178

[B41] Babalola S Tambashe BO Vondrasek C Parental factors and sexual risk-taking among young people in Cote d'Ivoire Afr J Reprod Heal 2005 9 1 49 65 10.2307/3583160 16104655

[B42] Kumi-Kyereme A Influence of social connectedness, communication and monitoring on adolescent sexual activity in Ghana Afr J Reprod Heal 2007 11 3 133 149 10.2307/25549736 PMC2367144 20698062

[B43] Amoran OE Fawole O Parental influence on reproductive health behavior of youths in Ibadan, Nigeria Afr JMed Med Sci 2008 37 1 21 27 18756851

[B44] Ajzen I Fishbein M Understanding Attitudes and Predicting Social Behaviour 1980 Englewood Cliffs: Prentice-Hall

[B45] Fisher JD Fisher WA Changing AIDS-risk behavior Psychol Bull 1992 111 3 455 474 1594721 10.1037/0033-2909.111.3.455

[B46] Catania J Kegeles S Coates T Toward an understanding of risk behavior: an AIDS risk reduction model (ARRM) Heal Educ Q 1990 17 53 72 10.1177/109019819001700107 2318652

[B47] Ajzen I The theory of planned behavior Organ Behav Hum Decis Process 1991 50 179 211 10.1016/0749-5978(91)90020-T

[B48] Bunton R Murphy S Bennett P Theories of behavioural change and their use in health promotion: some neglected areas Heal Educ Res 1991 6 2 153 162 10.1093/her/6.2.153

[B49] Hunt SM Martin CJ Health-related behavioural change - A test of a new model Psychol Heal 1988 2 209 230 10.1080/08870448808400352

[B50] Romer D Hornik R HIV education for youth: the importance of social consensus in behaviour change AIDS Care 1992 4 3 285 303 10.1080/09540129208253100 1525201

[B51] Michielsen K Concurrency and the limited effectiveness of behavioural interventions on sexual risk behavior of youth in sub-Saharan Africa AIDS 2010 24 13 2140 2142 20679762 10.1097/QAD.0b013e32833c8745

[B52] Appadurai A Rao V, Walton M The capacity to aspire: culture and the terms of recognition Culture and Public Action 2004 California: Stanford University Press 59 84

[B53] Douglas M Rao V, Walton M Traditional culture – let’s hear no more about it Culture and Public Action 2004 California: Stanford University Press 85 109

[B54] Wright S The Politicization of Culture 1997 Available from: http://lucy.ukc.ac.uk/rai/AnthToday/wright.html

[B55] Kuran T Rao V, Walton M Cultural obstacles to economic development Culture and Public Action 2004 California: Stanford University Press 115 137

[B56] Tawfik L Watkins SC Sex in Geneva, sex in Lilongwe, and sex in Balaka Soc Sci Med 2007 64 1090 1101 10.1016/j.socscimed.2006.10.002 17123678

[B57] Gupta G Structural approaches to HIV prevention Lancet 2008 372 764 775 10.1016/S0140-6736(08)60887-9 18687460

[B58] Sallis JF Owen N Fisher EB Glanz BKRKVK Ecological models of health behavior Health Behavior and Health Education 2008 San Fransisco: Jossey-Bass

[B59] Richard L Gauvin L Raine K Ecological models revisited: their uses and evolution in health promotion over two decades Ann Rev Public Health 2011 32 307 326 10.1146/annurev-publhealth-031210-101141 21219155

[B60] Bandura A Exercise of human agency through collective efficacy Curr Dir Psychol Sci 2000 9 3 75 78 10.1111/1467-8721.00064

[B61] Evans C Lambert H The limits of behaviour change theory: condom use and contexts of HIV risk in the Kolkata sex industry Cult Heal Sex 2008 10 1 27 41 10.1080/13691050701561393 18038279

[B62] Coates TJ Richter L Caceres C Behavioral strategies to reduce HIV transmission Lancet 2008 372 9639 669 684 10.1016/S0140-6736(08)60886-7 18687459 PMC2702246

[B63] Hankins C de Zalduondo B Combination prevention AIDS 2010 24 Supp 4 S70 S80 21042055 10.1097/01.aids.0000390709.04255.fd

[B64] Cooke B Kothari U Participation, the new tyranny? 2001 London: Zed

[B65] Mosse D Is good policy unimplementable? Reflections on the ethnography of aid policy and practice Dev Chang 2004 35 4 639 671 10.1111/j.0012-155X.2004.00374.x

[B66] Wilson D Partner reduction and the prevention of HIV/AIDS BMJ 2004 328 848 849 10.1136/bmj.328.7444.848 15073053 PMC387465

[B67] Panter-Brick C Culturally compelling strategies for behavior change: a social ecology model and case study in malaria prevention Soc Sci Med 2006 62 2810 2825 10.1016/j.socscimed.2005.10.009 16352385

[B68] Bastien S Reflecting and shaping the discourse: the role of music in AIDS communication in Tanzania Soc Sci Med 2009 68 7 1357 1360 10.1016/j.socscimed.2009.01.030 19232806

[B69] Department for International Development Cash Transfers Evidence Paper 2011 London: Department for International Development

[B70] Muyinda H Harnessing the senga institution of adolescent sex education for the control of HIV and STDs in rural Uganda AIDS Care 2003 15 2 159 167 10.1080/0954012031000068308 12856337

[B71] Barker D Ricardo C Young Men and the Construction of Masculinity in Sub-Saharan Africa. Social Development Papers No. 26 2005 Washington: The World Bank

[B72] UNAIDS AIDS Epidemic Update 2007 Geneva: Joint UN Programme on HIV/AIDS

[B73] Mavedzenge SMN Doyle AM Ross DA HIV prevention in young people in sub-Saharan Africa: a systematic review J Adolesc Heal 2011 49 6 568 586 10.1016/j.jadohealth.2011.02.007 22098767

[B74] Karim QA Effectiveness and safety of tenofovir gel for the prevention of HIV infection in women Science 2010 1168 1174 10.1126/science.1193748 PMC3001187 20643915

[B75] Ross DA Dick B Ferguson J Preventing HIV/AIDS in Young People: A Systematic Review of the Evidence from Developing Countries. WHo Technical Report Series 938 2006 Geneva: World Health Organisation 16921915

[B76] Vandenhoudt H Evaluation of a U.S. evidence-based parenting intervention for rural western Kenya AIDS Educ Prev 2010 22 4 328 343 10.1521/aeap.2010.22.4.328 20707693

